# Hexatetra-Carbon: A Novel Two-Dimensional Semiconductor Allotrope of Carbon

**DOI:** 10.3390/computation10020019

**Published:** 2022-01-25

**Authors:** Mosayeb Naseri, Jaafar Jalilian, Dennis R. Salahub, Maicon Pierre Lourenço, Ghasem Rezaei

**Affiliations:** 1 Department of Physics, Kermanshah Branch, Islamic Azad University, Kermanshah 67189-97551, Iran; mosayeb.naseri@ucalgary.ca; 2 CMS—Center for Molecular Simulation, Department of Chemistry, Department of Physics and Astronomy, IQST—Institute for Quantum Science and Technology, Quantum Alberta, University of Calgary, 2500 University Drive NW, Calgary, AB T2N 1N4, Canada; 3 Department of Physics, College of Sciences, Yasouj University, Yasouj 75918-74934, Iran; jaafarjalilian@gmail.com (J.J.); grezaei@yu.ac.ir (G.R.); 4 Departamento de Química e Física, Centro de Ciências Exatas, Naturais e da Saúde (CCENS), Universidade Federal do Espírito Santo, Alegre, Vitória 29500-000, Brazil; maiconpl01@gmail.com

**Keywords:** density functional theory, hexatetra-carbon, electrical properties

## Abstract

Employing first-principles calculations based on density functional theory (DFT), we designed a novel two-dimensional (2D) elemental monolayer allotrope of carbon called hexatetra-carbon. In the hexatetra-carbon structure, each carbon atom bonds with its four neighboring atoms in a 2D double layer crystal structure, which is formed by a network of carbon hexagonal prisms. Based on our calculations, it is found that hexatetra-carbon exhibits a good structural stability as confirmed by its rather high calculated cohesive energy −6.86 eV/atom, and the absence of imaginary phonon modes in its phonon dispersion spectra. Moreover, compared with its hexagonal counterpart, i.e., graphene, which is a gapless material, our designed hexatetra-carbon is a semiconductor with an indirect band gap of 2.20 eV. Furthermore, with a deeper look at the hexatetra-carbon, one finds that this novel monolayer may be obtained from bilayer graphene under external mechanical strain conditions. As a semiconductor with a moderate band gap in the visible light range, once synthesized, hexatetra-carbon would show promising applications in new opto-electronics technologies.

## 1. Introduction

The discovery of the interesting behavior of graphene [1] has motivated further theoretical and experimental investigations in order to find possible new stable two-dimensional (2D) materials, especially mono-elemental 2D monolayer materials [2,3,4,5,6,7]. As carbon exhibits a large number of different allotropes, such as graphite, diamond, C_60_ fullerene [8], nanotube [9], carbon nano-cone [10], and nanochain [11], 2D carbon mono-elemental monolayer materials beyond graphene have attracted significant attention from both theoretical and experimental fields of study. However, the electronic structure of graphene limits its application in designing electronic nano-devices due to its semi-metallic gapless nature. Therefore, finding new 2D mono-elemental monolayer materials with a semiconductor behavior is technologically important.

In recent years, the possibility of free standing 2D carbon allotropes beyond graphene has been explored. In addition, a number of new carbon allotrope monolayers has been proposed [12,13,14,15]. Although these 2D materials, such as graphdiyne [12], penta-graphene [13], and phagraphene [14] are metastable compared with graphene, only a few have been successfully synthesized. Moreover, these new 2D carbon monolayers exhibit very interesting properties, such as anisotropic Dirac cones, inherent ferromagnetism, high catalytic activity, and potential superconductivity related to the high density of states at the Fermi level. This demonstrates that structural properties and the crystal configuration of 2D carbon allotropes effectively influence the electronic, optical, and other chemo-physical properties of these materials [12,13,14,15,16,17,18,19]. For instance, graphdiyne [12] is a predicted flat one-atom-thick allotrope of carbon with a Dirac cone in its band structure, in which the Dirac points are located at the *K* point. On the other hand, penta-graphene is a new 2D carbon allotrope with semiconducting properties that stabilizes in a buckled structure composed entirely of carbon pentagons, and resembles the Cairo pentagonal tiling with an intrinsic indirect band gap of about 3.25 eV [13]. Moreover, phagraphene, which is a monolayer sheet of carbon with a structure composed of 5-6-7 carbon rings has distorted Dirac cones [14].

On the other hand, carbon nanostructures have tetragons, pentagons, and hexagons as their main basic building blocks. For instance, cubane (C_8_H_8_) is a synthetic hydrocarbon molecule formed by eight carbon atoms positioned at the corner of a cube. It is attached to its three neighboring carbon atoms and a hydrogen atom with tetragonal top and side views [20], 2D graphene, and 3D graphite, which are formed by carbon hexagons. In addition, penta-graphene consists entirely of pentagons of carbon atoms. Moreover, the C_60_ molecule is formed by 12 pentagons, which are separated by 20 hexagons with a soccer ball shape [8]. 

In this paper, using first-principles calculations based on DFT, we propose a novel 2D elemental monolayer allotrope of carbon, which is called hexatetra-carbon due to its hexagonal and tetragonal top and side views. In a crystal network of the newly proposed 2D carbon allotrope, each carbon atom binds with its four neighboring atoms in a 2D double layer crystal structure, which is formed by hexagonal prisms. The meta-stability of our newly designed monolayer is shown by the cohesive energy and phonon calculations. Furthermore, evaluating the electrical properties of hexatetra-carbon shows that it exhibits a semiconductor behavior with a moderate band gap. 

The current paper is organized as follows: The computational method used in our study is presented in Section 2. In Section 3, the details of the structural properties are outlined and the stability of the proposed structure is discussed. The electrical characteristics of the designed hexatetra-carbon monolayer and its potential applications are described in Section 4. Finally, in Section 5, the paper is concluded.

## 2. Computational Methods

To obtain accurate structural and electrical properties of the proposed 2D material, the full-potential linearized-augmented plane wave (FP-LAPW) scheme was utilized [21]. This scheme is based on the DFT implemented in the WIEN2k computational package [22], in which the generalized gradient approximation (GGA) parameterized by Perdew–Burke–Ernzerhof (PBE) was used [23]. Moreover, since the GGA method underestimates band gaps, to obtain more reliable band gaps, the screened short range hybrid functional exchange correlation implemented in the WIEN2k code [24] was employed for band gap calculations. Moreover, the Monkhorst–Pack scheme [25] was used to sample the Brillouin zone by a 24 × 24 × 1 k-mesh, where we chose RK_max_ = 7, Gmax = 14 Ry^1/2^, and l_max_ = 10. To avoid interlayer interactions, a large vacuum distance of 20 Å along the non-periodic direction was utilized. With regards to dynamic stability, an evaluation of all the calculations was conducted with the Quantum Espresso (QE) package [26]. Furthermore, the Martin–Troullier norm-conserving pseudopotential [27] was used to treat the core electrons, while the valence electronic wave functions were expanded using an energy cut-off of 80 Ry. However, for the investigation of structural properties of bilayer graphene, we considered the van der Waals correction in our calculations.

## 3. Structural Properties and the Stability of Hexatetra-Carbon Monolayer

**Figure 1:**
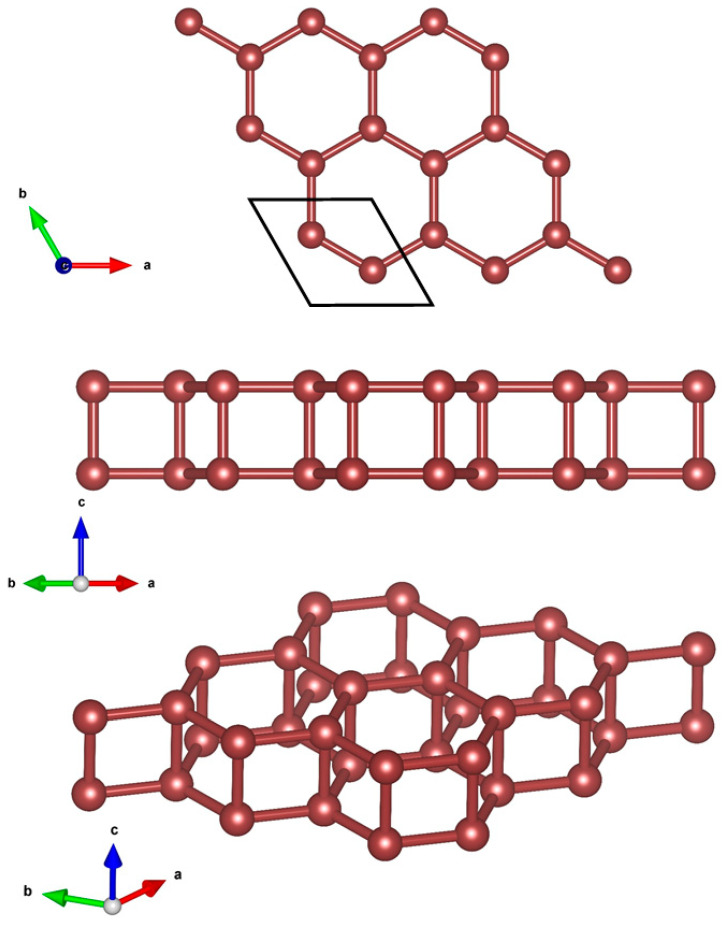
Crystal structure of 2D hexatetra-carbon from different views.

The design of the 2D hexatetra-carbon monolayer was initiated by examining the effect of vertical compressive strains on the structural, electronic, and optical properties of bilayer graphene in its AA-stacking configuration. Recently, several research works have reported the influence of strain on the different physical characteristics of bilayer graphene. Moreover, it is well-known that a crystal structure of a bilayer graphene stabilizes at the interlayer distance of about $h_{0}$ = 3.4 Å layer separation, in which the long-range van der Waals (vdW) interaction plays an important role in the structural properties of the materials. In this case, when evaluating the effect of vertical compressive strains on the different properties of AA-stacked bilayer graphene, with the aim of preserving the stability of the initial AA-stacked bilayer graphene, researchers mostly consider vertical strains that influence a maximum variation of 20% in interlayer separation, i.e., $h=h_{0}\pm 20\%\;h_{0}$ (in which the long-ranged van der Waals (vdW) interaction should be taken into account). In these conditions, not only the stability of the AA-stacked bilayer graphene is preserved, but also its crystal structure configuration is retained. However, one may be curious to know the effect of higher values of compressive vertical strains, in which the layer separation distance of h < 2.0 Å for the long-range van der Waals (vdW) interaction has no important effect. On this basis, we found a new unprecedented 2D allotrope of carbon called hexatetra-carbon during our structure searches. Figure 1 shows the optimized structure of our designed hexatetra-carbon monolayer from different views. A unit cell of hexatetra-carbon monolayer consists of four carbon atoms with optimized lattice constants of a = b = 2.65 Å. In a crystal network of the hexatetra-carbon, C atoms are distributed in two different exactly planar atomic planes with a vertical distance of about 1.55 Å. Specifically, each C atom of the hexatetra-carbon bonds to four neighboring C atoms to form a tetra-coordinated carbon structure, in which the C–C bond lengths are about 1.55 Å, longer than those in graphene (1.43 Å) and almost equal to the C1–C2 bond lengths in penta-graphene [13].

**Figure 2:**
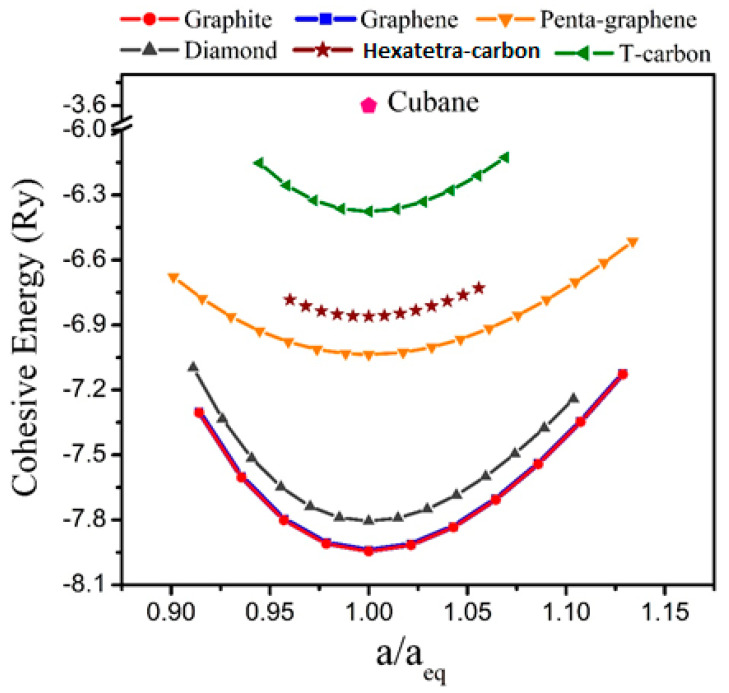
Cohesive energy of different carbon allotropes.

Furthermore, before an evaluation could be performed for the electrical properties and potential applications of the hexatetra-carbon monolayer, it is imperative to analyze the stability of the monolayer structure. This was carried out by first evaluating its energetic stability through a calculation of its cohesive energy, which is given by $E_{coh}=\frac{E_{hexatetra-carbon}-4E_{c}}{4}$ ($E_{c}$, $E_{hexatetra-carbon}$ are the total energies of a single C atom, and a unit cell of the hexatetra-carbon monolayer, respectively). Based on our calculation, the 2D hexatetra-carbon monolayer has a cohesive energy of about −6.86 eV/atom. For comparison, we also calculated the cohesive energy of graphene, penta-graphene, and T-carbon [28], which is a 3D carbon allotrope obtained by replacing each atom in diamond with a carbon tetrahedron at the same theoretical level. The values were about −8.01, −7.02, −6.34 eV/atom, respectively, which confirm that the proposed 2D hexatetra-carbon monolayer shows good energetic stability. Figure 2 shows a unit cell relative energy of the predicted hexatetra-carbon monolayer compared with different carbon allotropes under strain conditions.

**Figure 3:**
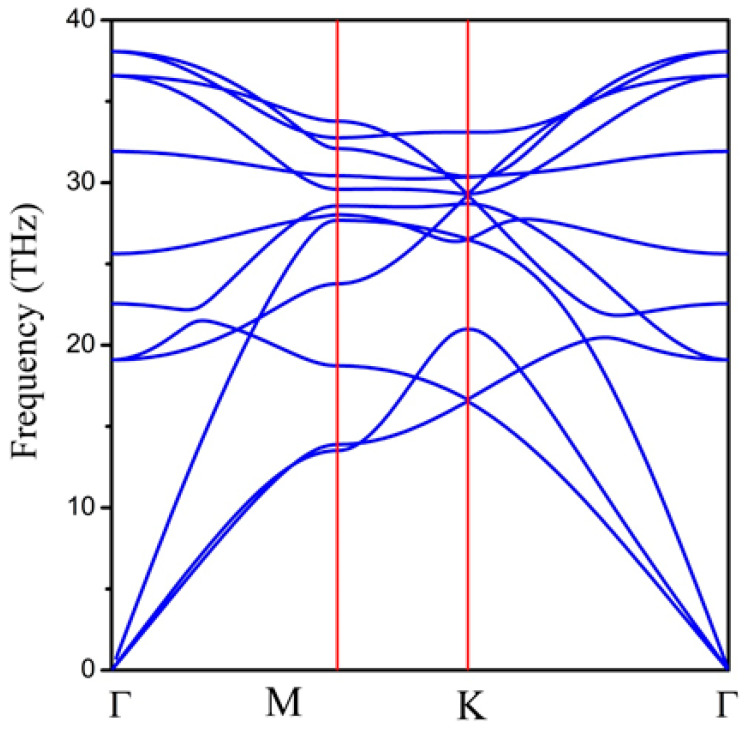
Phonon dispersion spectrum of 2D hexatetra-carbon monolayer.

Next, we examined the dynamic stability of the 2D hexatetra-carbon monolayer by calculating its phonon dispersion spectrum. As shown in Figure 3, there are no imaginary phonon modes in the whole Brillouin zone, indicating that the hexatetra-carbon is a local minimum on the potential energy surface and can be considered as a metastable allotrope of carbon compared with the other carbon allotropes. Specifically, we obtained the highest frequency of 1267 cm^−1^, which is higher than those obtained for silicon [29] (580 cm^−1^), MoS2 monolayer [30] (473 cm^−1^), and TiC monolayer [31] (810 cm^−1^). However, it is lower than the highest phonon frequency of graphene (about 1650 cm^−1^) and penta-graphene (about 1600 cm^−1^), indicating robust C–C bonds in the predicted monolayer.

From the above indications that the 2D hexatetra-carbon may be meta-stable, we next systematically study its mechanical properties. Using Young’s modulus (*Y*), Equation (1) can be calculated [32], where ε is the strain in the vicinity of the optimum lattice vector (ε = (*a* − a_0_)/a_0_) and *E* is the total energy. In addition, *V*_0_ is the equilibrium volume of the 2D material evaluated by $V_{0}=\frac{3\sqrt{3}d^{2}h}{2}$, where *d* is the adjacent carbon distance in the hexagon ring in the *a* and *b* plane, and *h* is the thickness of the 2D material along the *c* vector (Figure 1).

(1) $$Y=\frac{1}{V_{0}}{\left(\frac{\partial^{2}E}{\partial \varepsilon^{2}}\right)}_{\varepsilon =0}$$

In this work, the estimated Young’s modulus value for hexatetra-carbon is 1859.7 GPa. It is smaller than those estimated by Raman spectroscopy [33] for the single- and bilayer graphene, 2400 ± 400 GPa and 2000 ± 500 GPa, respectively. With regards to carbon fibers, the Young’s modulus value is 235–427 GPa [34]. Here, the value of hexatetra-graphene is greater than those obtained for other materials, such as imogolites (∼320–370 GPa) [35] and GaS (∼270 GPa) [36], as well as the nanotubes and MoS_2_ monolayer (265 ± 13 GPa), which are similar to the bilayer [37].

## 4. Electronic Properties

**Figure 4:**
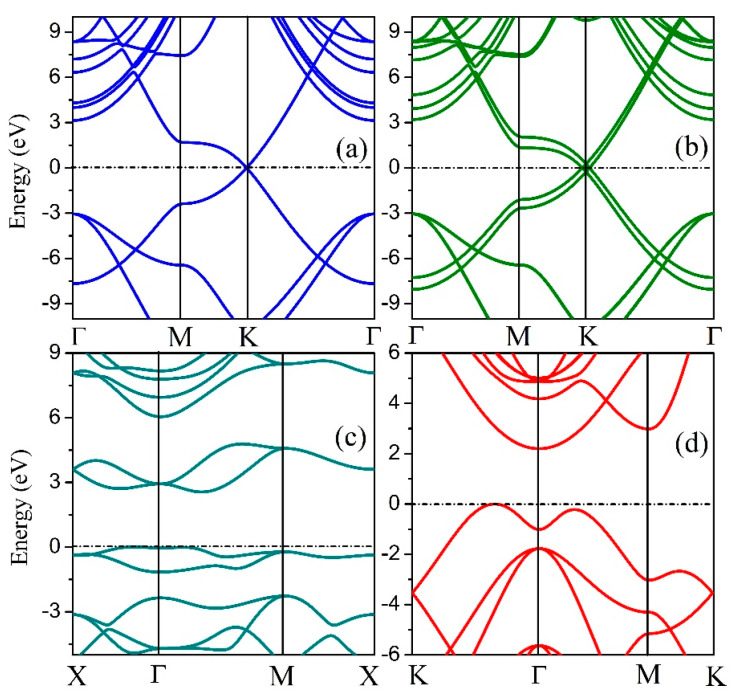
Band structures of (**a**) graphene, (**b**) bilayer graphene, (**c**) penta-graphene, and (**d**) hexatetra-carbon.

To analyze the electrical properties of the 2D hexatetra-carbon monolayer, we calculated the band structure of the designed 2D monolayer. As shown in Figure 4, an indirect bandgap of about 2.20 eV, which is calculated using the hybrid functional level of theory can be seen in the band structure. Moreover, the valence band maximum (VBM) of this 2D monolayer material is located between K and Γ points, while the conduction band minimum (CBM) is located at the Γ point. Therefore, the 2D hexatetra-carbon monolayer, which is a semiconductor with a moderate band gap, is not similar to the monolayer and bilayer graphene due to the fact that they are both semi-metals. 

**Figure 5:**
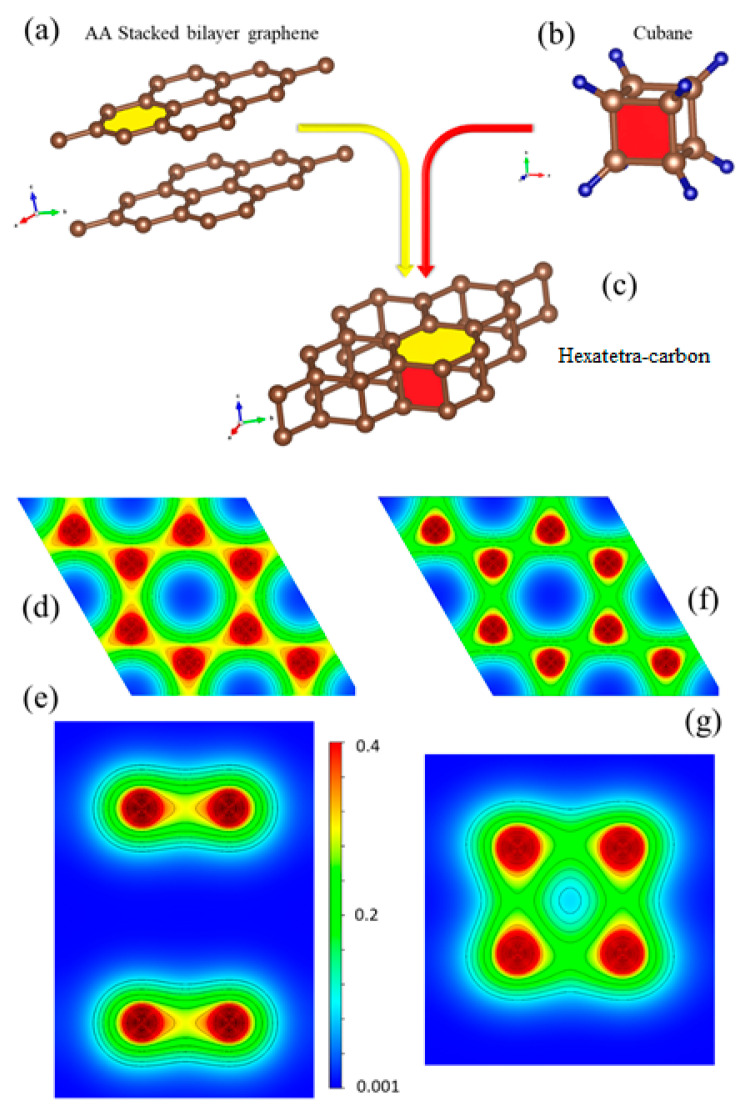
Crystal structure of (**a**) AA-stacked bilayer graphene, (**b**) cubane molecule, and (**c**) hexatetra-carbon (**d**,**e**). Top and side views of valence charge density distribution for AA-stacked graphene and (**f**,**g**) for hexatetra-carbon obtained by WIEN2K code [21].

To gain a deeper insight into the bonding nature of hexatetra-carbon and its structural analogy with respect to AA-stacked graphene, we investigated the 2D valence charge density distribution of these two nanostructures (see Figure 5d–g). The crystal structure of AA-stacked bilayer graphene, the cubane molecule, and hexatetra-carbon are shown in Figure 5a–c. It is clear that the hexatetra-carbon structure has a hexagonal face similar to graphene (xy plane). In addition, interlayer bonds along the z direction between the two graphene monolayers show a tetragonal side view, which is similar to the cubane molecule.

As shown in Figure 5d, there are three sp^2^ sigma covalent bonds (1.43 Å) between the C–C atoms in each monolayer of AA-stacked bilayer graphene, as well as a weak p_z_-p_z_ interaction between the monolayers. In comparison, the in-plane bond length of C–C for hexatetra-carbon is 1.56 Å, which is longer than those obtained in AA-stacked bilayer graphene, i.e., the C–C in-plane orbital overlap decreases for the hexatetra-carbon (see Figure 5f). Therefore, to retain its structural stability, the hexatetra-carbon nanostructure compensates this orbital variation by creating interlayer sigma bonds between the neighboring carbons, which are located in the different planes. Moreover, these interlayer bonds would restrict p_z_, resulting in the semiconducting nature of 2D hexatetra-carbon.

**Figure 6:**
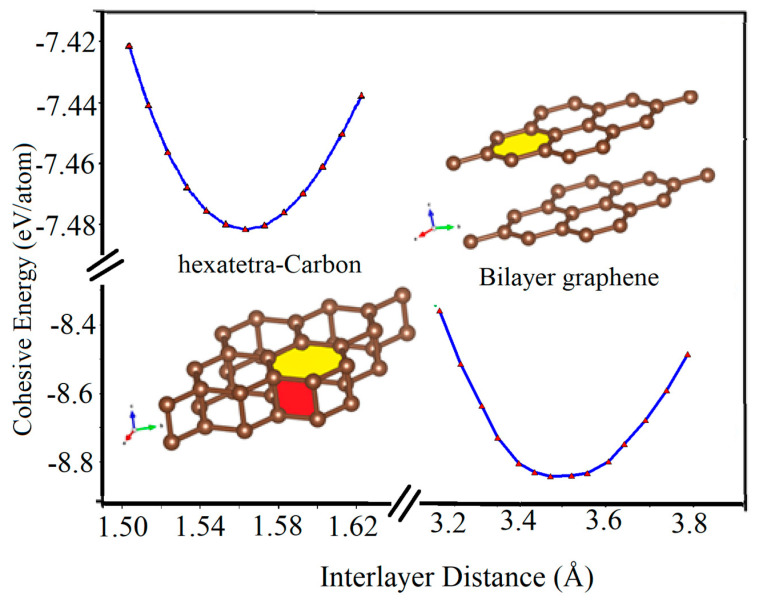
Cohesive energy of AA-stacked bilayer graphene and hexatetra-carbon versus interlayer distance calculated by Quantum Espresso (due to the interruption in accessing WIEN2k while following up on a reviewer’s comment, we have used Quantum Espresso for Figure 6).

Furthermore, we calculated the variation of the cohesive energy of two graphene sheets as they approach each other. As seen in Figure 6, when a graphene sheet moves towards another fixed graphene sheet, they tend to stabilize at the vertical distance of about 3.45 Å and form the AA-stacked bilayer graphene structure, which is the most stable structure in this situation. However, by applying an additional external vertical mechanical strain to the AA-stacked bilayer graphene through decreasing its interlayer distance, its lattice parameter increases. In addition, another local energy minimum occurs for the two graphene sheets with a vertical interlayer distance of about 1.55 Å. In this case, the hexatetra-carbon includes four carbon atoms in its unit cell, and the lattice parameter of about 2.65 Å structure is formed. In other words, by applying the external vertical strain, a type of phase transition occurs.

## 5. Summary

In conclusion, utilizing first-principles calculations based on DFT, we proposed a new 2D monolayer crystalline allotrope of carbon called hexatetra-carbon, which shows a semiconducting nature with a moderate band gap. The stability of 2D hexatetra-carbon monolayer is confirmed by its rather high cohesive energy, as well as the absence of imaginary phonon modes in its phonon dispersion spectrum. Comparing the cohesive energy of the hexatetra-carbon monolayer with those obtained for other 2D allotropes confirms its high bonding properties. Moreover, our analysis on the charge densities of both bilayer graphene and hexatetra-carbon monolayer indicates that by applying a vertical strain on bilayer graphene, the hexatetra-carbon structure can be obtained. Therefore, one may be optimistic that the 2D hexatetra-carbon monolayer can be experimentally achieved in the foreseeable future.

## Data Availability

Not applicable.
